# Pathological Study of a *FMR1* Premutation Carrier With Progressive Supranuclear Palsy

**DOI:** 10.3389/fgene.2018.00317

**Published:** 2018-08-15

**Authors:** Martin Paucar, Inger Nennesmo, Per Svenningsson

**Affiliations:** ^1^Department of Neurology and Clinical Neuroscience, Karolinska Institutet, Karolinska University Hospital, Stockholm, Sweden; ^2^Department of Pathology, Karolinska Institutet, Karolinska University Hospital, Stockholm, Sweden

**Keywords:** FXTAS, CBS, PSP, *FMR1*, RAN translation

## Abstract

Dual pathology in fragile X mental retardation 1 (*FMR1)* premutation carriers and fragile X–associated tremor/ataxia syndrome (FXTAS) patients is an emerging phenomenon. Although it includes atypical parkinsonism, neuropathological confirmation is very scarce. Here, we describe neuropathological findings for a female who suffered a severe parkinsonian syndrome with apraxia and supranuclear palsy. She died at the age of 50, six years after the initial diagnosis. Prominent neuronal loss was found in the pallidum, subthalamic nucleus, and tectum, but the loss of Purkinje cells was rather mild. Intranuclear inclusions containing ubiquitin and FMRpolyglycine, a pathological hallmark of FXTAS, were detected in neurons and astrocytes. However, this inclusion pathology was overshadowed by a very prominent four repeat tau accumulation in tufted astrocytes, oligodendroglial coiled bodies, thread structures, and neurons. This is, to best of our knowledge, the first report describing a pathologically confirmed progressive supranuclear palsy – corticobasal syndrome (PSP-CBS) variant case in a *FMR1* premutation carrier.

## Introduction

The fragile X–associated tremor/ataxia syndrome (FXTAS) is an adult-onset, progressive neurodegenerative disorder that affects individuals with a premutation (between 55 and 200 CGG repeats) in the fragile X mental retardation 1 (*FMR1*) gene (Hagerman et al., 2001; Jacquemont et al., 2003). Forty to seventy five percent of males and 16–20% of females with *FMR1* premutations develop FXTAS (Hagerman and Hagerman, 2016). Although the main clinical features of FXTAS are cerebellar gait ataxia and action tremor (Apartis et al., 2012; Hagerman and Hagerman, 2013), a subset of FXTAS patients suffer from nigrostriatal dysfunction and parkinsonism (Fraint et al., 2014; De Pablo-Fernandez et al., 2015; Paucar et al., 2016). Diagnoses of parkinsonian syndromes are based on clinical criteria, in which a definite diagnosis is achieved by neuropathological assessment. Here, we report histological findings of a patient with a *FMR1* premutation, who had a severe dopamine deficiency, according to [^123^I] ioflupane SPECT, and showed clinical signs compatible to the progressive supranuclear palsy – corticobasal syndrome variant (PSP-CBS) as we have previously reported (Paucar et al., 2016).

## Materials and Methods

This report was made within the frame of a study approved by the local ethics committee; the patient and her relatives have signed informed consent. Tissue for microscopy was collected according to a standard protocol for neurodegenerative disorders and included cortical areas from all lobes of the cerebrum, hippocampus, basal ganglia, thalamus, mesencephalon, pons, medulla oblongata and cerebellum. Five micrometer thick paraffin sections from all regions were stained with haematoxylin-eosin, luxol fast blue and modified Bielschowsky silver staining. Immunostainings with antibodies against hyperphosphorylated tau (AT8; Thermo Scientific) were made on sections from different cortical regions, basal ganglia, mesencephalon, pons, and cerebellum. Sections from different cortical regions, hippocampus, basal ganglia and cerebellum were also stained with antibodies against ubiquitin (Merck Millipore, Burlington, MA, United States). Sections from hippocampus were stained with antibodies against FMRpolyglycine (FMRpolyG) (clone 9FM-1B7; Merck Millipore, Burlington, MA, United States). Antibodies against three repeat (3R) and four repeat (4R) tau (both from Merck Millipore, Burlington, MA, United States) were used on sections from the frontal lobe. Immunostainings were performed on five μm thick paraffin sections on superfrost slides in a Bond immunostainer.

## Results

### Phenomenology and Clinical Investigations

The patient who we have previously reported (Paucar et al., 2016), was a female harboring 82 CGG repeats in the *FMR1* gene. Her past medical record included premature ovarian failure (POF), the age of onset for motor symptoms was 44, and total disease duration six years. The phenotype consisted of severe and fast progressing parkinsonism, cognitive decline, apraxia, tachyphemia, echolalia and supranuclear palsy. Cognitive domains related to language, executive functions, attention, and memory were progressively impaired. Her saccades became slow and had increased initiation latency. The patient became wheelchair-bound, incontinent and developed akinetic mutism, and dystonia. These symptoms were unresponsive to L-dopa treatment or inhibition of MAO B or choline esterase and were compatible to PSP-CBS. MRI revealed mild atrophy in the mesencephalon, cerebellum and cortex. [^123^I] ioflupane SPECT demonstrated a major loss of the dopamine transporter in the basal ganglia. FDG-PET showed bilateral hypometabolism in frontal cortical lobes. Neurofilament light chain in the cerebrospinal fluid was slightly elevated, but beta-amyloid, tau and phospho-tau levels were normal. The patient died during sleep and an autopsy was performed.

### Neuropathological Examination

**FIGURE 1:**
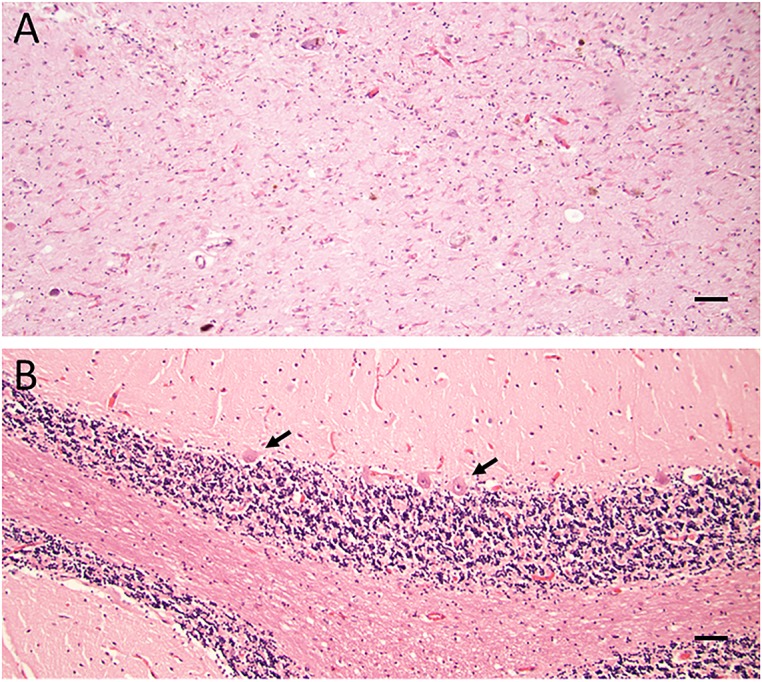
**(A,B)** Haematoxylin-eosin staining showing severe loss of neuromelanin-containing neurons in substantia nigra and marked astrogliosis **(A)**. Reduction in the number of Purkinje cells (arrows) in cerebellum **(B)**. Scale bars: 100 μm.

**FIGURE 2:**
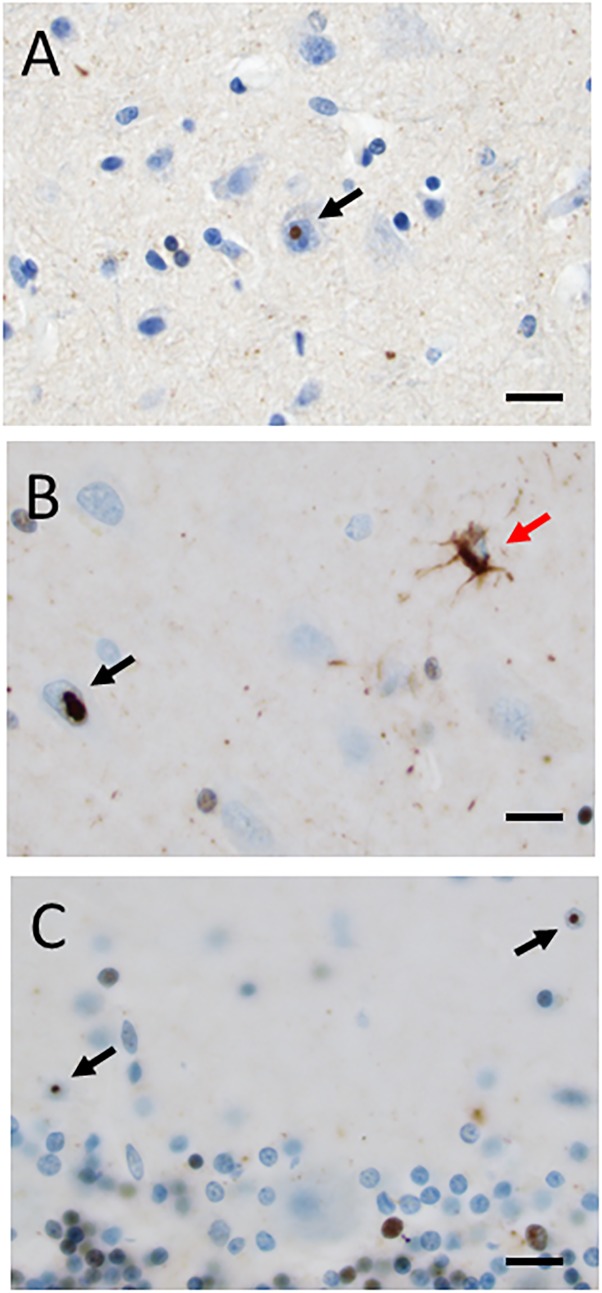
**(A–C)** Ubiquitin-positive intranuclear inclusions (black arrows) in neurons in frontal cortex **(A)**, hippocampus **(B)**, and cerebellum **(C)**. A red arrow in **(B)** indicates an ubiquitin-positive tufted astrocyte. Scale bars: 25 μm.

**FIGURE 3:**
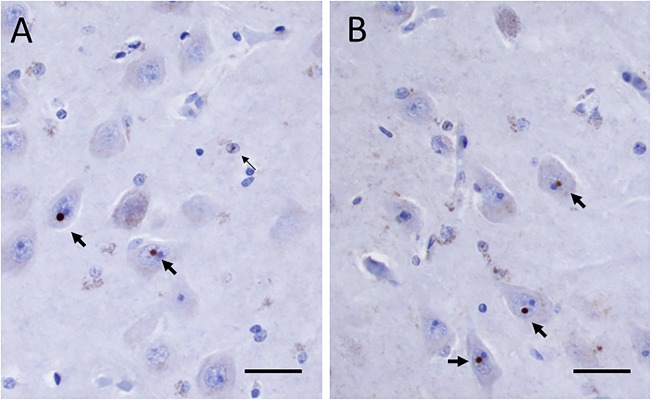
**(A,B)** FMRpolyG positive inclusions in neurons (thick arrows) and in astrocyte-like cell (thin arrow in panel **A**) in the CA4 region of the hippocampus. Scale bars: 25 μm.

**FIGURE 4:**
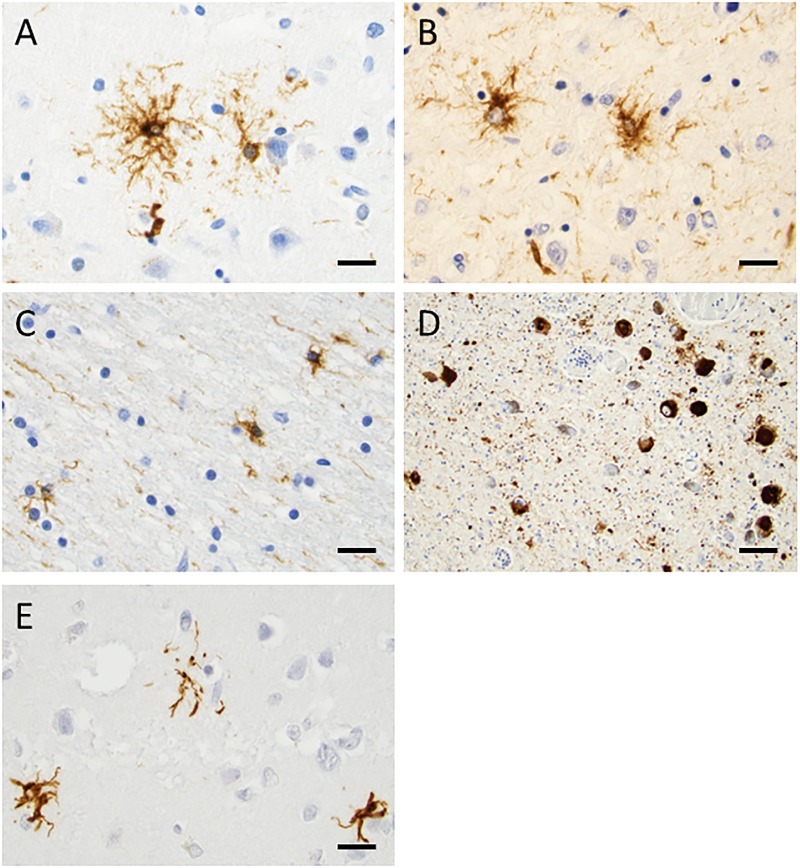
Tau staining with the AT8 antibody, demonstrating phosphorylated tau. In frontal cortex **(A)** and tectum **(B)**, tufted astrocytes were frequently found. In the white matter, there were many oligodendroglial coiled bodies along with thread-like structures **(C)**. Several neurons in locus coeruleus showed dense tau staining **(D)**. 4R tau staining in tufted astrocytes in the frontal cortex was evident **(E)**. Scale bars: 25 μm.

The formaldehyde-fixed brain weighed 1120 g, of which the cerebellum weighed 120 g. The frontal gyri appeared to be somewhat smaller than expected, and the thickness of the cortex was slightly reduced in the frontal lobes. The ventricles were of ordinary size. The substantia nigra was pale but without focal lesions. Microscopically, there was a severe reduction in neuromelanin-containing neurons with astrogliosis in the substantia nigra (**Figure 1A**). There was only a mild loss of pigmented neurons within the locus ceruleus in pons. Prominent neuronal loss and astrogliosis was evident in the pallidum, subthalamic nucleus and tectum. Mild loss of Purkinje cells in the cerebellar cortex was found (**Figure 1B**). In some neurons, small ubiquitin-positive intranuclear inclusions were present in the frontal lobe cortex (**Figure 2A**) and hippocampus (**Figure 2B**) as well as in the basal ganglia. Intranuclear inclusions were detected in neurons of the molecular cell layer of the cerebellum (**Figure 2C**). **Figure 2B** also illustrates an ubiquitin-positive tufted astrocyte. Inclusions containing FMRpolyG were prevalent in neurons of the hippocampus, especially in the CA4 region (**Figures 3A,B**). Occasionally, astrocyte-like cells also contained FMRpolyG positive inclusions (**Figure 3A**; thin arrow). The tau pathology was widespread, and tufted astrocytes were present in cortical regions, especially in the frontal lobe (**Figure 4A**). The tau pathology was also evident in the basal ganglia and tectum (**Figure 4B**). Numerous oligodendroglial coiled bodies were seen in the white matter (**Figure 4C**). In both gray and white matter, there were several tau-positive thread structures (**Figures 4B,C**). Tau-immunopositive neurons were especially numerous in the pons, including the locus ceruleus (**Figure 4D**). On sections stained with antibodies against 3R or 4R tau, only 4R tau positivity was found in these structures. **Figure 4E** illustrates tufted astrocytes stained for 4R tau in frontal cortex.

To compare tau and nuclear inclusion pathologies, tau positive tufted astrocytes and ubiquitin inclusions were counted in four ×20 objective microscopic fields of view. In frontal and temporal cortices, 65 and 44 tau-positive tufted astrocytes were found, respectively. In the same cortical regions, two and four ubiquitin-positive inclusions were detected, respectively. No tau immunoreactivity was seen in the cerebellar cortex whereas six ubiquitin-positive inclusions were detected in the molecular layer.

## Discussion

Tau-pathology, specifically 4R tau, was identified in tufted astrocytes, oligodendroglial coiled bodies, thread structures and neurons. Thus, the character and distribution of the pathological abnormalities in the present case correspond to PSP (Dickson et al., 2010). To best of our knowledge, this is the first report presenting a typical PSP pathology in a *FMR1* premutation carrier. It is worth to mention that the patient’s father fulfills not only the criteria for FXTAS, but also displays the hummingbird sign, a hallmark of PSP, as well as dopamine deficiency on DAT scan (Paucar et al., 2016).

We also found intranuclear ubiquitin-positive inclusions in the cerebral and cerebellar cortex as well as in the hippocampus and basal ganglia, which were consistent with FXTAS pathology. The presence of intranuclear inclusions in neurons and astrocytes is the pathological hallmark of FXTAS (Greco et al., 2002, 2006; Tassone et al., 2004). The inclusions are, in most cases, single and found throughout the brain with the highest abundance in the hippocampus. The intranuclear inclusions contain ubiquitin, heat-shock proteins, RNA-binding protein hnRNP A2 and *FMR1* mRNA to a less degree (Iwahashi et al., 2006). Recently, FMRpolyG was identified in ubiquitin-positive inclusions in a mouse model of FXTAS and brains of FXTAS patients (frontal cortex and hippocampus) and induced pluripotent cells generated from FXTAS patients (Todd et al., 2013; Sellier et al., 2017). Accordingly, we found FMRpolyG-positive inclusions in the hippocampus. FMRpolyG is the result of repeat-associated non-ATG (RAN) translation phenomenon. RAN translation has been found in other nucleotide expansion disorders (e.g., spinocerebellar ataxia 8 and myotonic dystrophy type 1**)** (Zu et al., 2011). RAN translation generates toxic peptides, but what regulates this process is not completely understood. The sole expression of the FMRpolyG is toxic in cell cultures and in *Drosophila* but only translation of expanded CGG repeats into FMRpolyG causes motor abnormalities in mice (Sellier et al., 2017). Full CGG expansion in *FMR1* leads to hypermethylation and silencing of this gene, while 2–8 fold elevation of *FMR1* mRNA levels in premutation carriers only slightly reduce the expression of the gene product. Other than RAN translation, RNA toxicity induced by sequestration of transcription factors is another major underlying mechanism of disease for FXTAS (Glineburg et al., 2018).

Dual pathology in FXTAS includes disparate neurological conditions, such as chronic fatigue syndrome, multiple sclerosis, inclusion body myositis and atypical parkinsonism among many others (Greco et al., 2006; Tassone et al., 2012; Fraint et al., 2014; De Pablo-Fernandez et al., 2015; Kaub-Wittemer et al., 2017; Lechpammer et al., 2017). Neuropathological evidence of comorbidity among *FMR1* premutation carriers is scarce. However, there were four cases reported to have Alzheimer’s disease, one with dementia with Lewy bodies, and four with Parkinson’s disease (Greco et al., 2006; Tassone et al., 2012; De Pablo-Fernandez et al., 2015; summarized in **Table 1**).

**Table 1:** Summary of *FMR1* premutation carriers with pathologically-confirmed concomitant proteinopathy.

Phenotype	Number of cases (sex)	Concomitant proteinopathy	Reference
FXTAS and dementia	4	Alzheimer’s disease (3)	Tassone et al., 2012
	(All female)	Dementia with Lewy bodies (1)	
FXTAS	3	Parkinson’s disease	Greco et al., 2006;
	(2 males, 1 female)		Tassone et al., 2012^∗^;
			De Pablo-Fernandez et al., 2015
PSP	1	Parkinson’s disease	De Pablo-Fernandez et al., 2015
	(male)		
PSP	1	PSP	This work
	(female)		

^∗^*These authors reported also concomitant multiple sclerosis*.

Fragile X–associated tremor/ataxia syndrome and PSP are associated with a wide phenotypic variability and overlapping clinical features. Up to 42% of PSP patients suffer from some form of tremor (Fujioka et al., 2016). There is also a PSP subtype with predominant cerebellar ataxia (Kanazawa et al., 2013). Vertical gaze palsy, proceeded by slowed vertical saccades, are typical signs for PSP. These eye movement abnormalities along with dysmetric saccades, transient endgaze nystagmus, and square wave jerks have been observed in FXTAS patients (Fraint et al., 2014).

The clinical PSP cases reported in a FXTAS cohort in the United States await pathological confirmation (Fraint et al., 2014). Moreover, larger studies are needed in order to assess whether the *FMR1* premutation and/or other epigenetic factors are related to predispose tauo- and synucleinopathies. If an association can be confirmed, then elucidating the underlying mechanism(s) for this comorbidity should be a priority in future research.

## Concluding Remarks

This is the first presentation of characteristic PSP pathology in a *FMR1* premutation carrier further emphasizing the occurrence of dual pathology in FXTAS cases.

## Ethics Statement

The patient had given oral and written consent for this work. The study was approved by the local ethical committee (2011/500-31/1).

## Author Contributions

MP and PS involved in patient workup. IN performed the pathological examination. MP, IN, and PS drafted the manuscript, approved the final manuscript, and agreed to be accountable of the work.

## Conflict of Interest Statement

The authors declare that the research was conducted in the absence of any commercial or financial relationships that could be construed as a potential conflict of interest. The handling Editor and reviewer RH declared their involvement as co-editors in the Research Topic, and confirm the absence of any other collaboration.
